# Comparison of T-POD and SAM Pelvic Sling II and the influence of attachment level in the initial management of unstable pelvic type C injuries – a cadaveric study

**DOI:** 10.1186/s12245-024-00610-8

**Published:** 2024-03-04

**Authors:** Maxim Privalov, Malte Junge, Matthias Karl Jung, Sven Yves Vetter, Jochen Franke, Svetlana Hetjens, Paul Alfred Grützner, Holger Stadthalter, Niko R.E Schneider

**Affiliations:** 1 Department for Trauma and Orthopaedic Surgery, BG Klinik Ludwigshafen, Ludwig-Guttmann-Str. 13, 67071 Ludwigshafen, Germany; 2 Department for Trauma and Orthopaedic Surgery, Tauernklinikum, Paracelsusstraße 8, Zell am See, 5700 Austria; 3 Department of Medical Statistics, Biomathematics and Data Processing, Universitätsklinikum Mannheim, Theodor-Kutzer-Ufer 1-3, 68167 Mannheim, Germany; 4 Friedrich-Alexander-University Erlangen-Nürnberg, Krankenhaus-Str. 12, 91054 Erlangen, Germany; 5 Department for Trauma and Orthopaedic Surgery, AUVA Unfallkrankenhaus, Dr.-Franz-Rehrl-Platz 5, Salzburg, 5010 Austria; 6 Department of Anesthesiology, Operative Intensive Care Medicine and Pain Therapy, University Hospital of Gießen and Marburg, Gießen, Germany; 7 Medical Faculty Heidelberg, Department of Anesthesiology, Heidelberg University, Heidelberg, Germany

**Keywords:** Pelvic fracture, Unstable pelvic Injury, Pelvic binder, T-POD, SAM pelvic Sling II, Pelvic stabilization

## Abstract

**Background:**

Type C pelvic fractures (AO/OTA) are severe injuries that frequently lead to bleeding and hemodynamic instability. Pelvic binders play a crucial role in their initial management. Placement at the correct level in the prehospital setting is challenging. The aim of this study was to compare two pelvic binders regarding their effectiveness in reducing intrapelvic volume and increasing intrapelvic pressure in patients with type C pelvic fractures (AO/OTA) when applied at three different levels.

**Methods:**

Rotationally and vertically unstable pelvic injuries (AO/OTA classification 61-C1.1) were produced in five fresh-frozen human cadaveric specimens. Intrapelvic volume, vesical pressure and compression pressure within the pubic symphysis and the sacroiliac joint were measured when applying a SAM Pelvic Sling II and a T-POD at the level of the greater trochanter as well as levels higher and lower than recommended.

**Results:**

Comparison of the two pelvic binders positioned at the recommended level (greater trochanter) showed no significant difference in volume reduction (13.85 ± 31.37 cm^3^, *p* = 0.442), however, increase in vesical pressure was significantly higher when using the T-POD (5.80 ± 3.27 cmH_2_O, *p* = 0.017). When positioned at the level of the iliac crest, vesical pressure increase and intrapelvic volume reduction were significantly greater with the T-POD (14.00 ± 8.57 cmH_2_O, *p* = 0.022 and 10.45 ± 5.45 cm^3^, *p* = 0.031 respectively). Application of the SAM Pelvic Sling II below the greater trochanter led to a significantly greater decrease in volume (-32.26 ± 7.52 cm^3^, *p* = 0.003) than the T-POD. Comparison of the recommended attachment level with incorrect positioning led to no significant differences for the T-POD, while the SAM Pelvic Sling II achieved a significantly lower volume reduction when placed at the iliac crest (40.15 ± 14.57 cm^3^, *p* = 0.012) and a significantly lower increase in vesical pressure when applied below the greater trochanter (3.40 ± 1.52 cmH_2_O, *p* = 0.007).

**Conclusion:**

Direct comparison of the two pelvic binders showed that the T-POD achieved significantly greater results when applied at the recommended level and was less susceptible to incorrect positioning. These outcomes support the preferred use of the T-POD for prehospital emergency pelvic stabilisation.

## Introduction

Pelvic fractures are among the most serious injuries to the human skeletal system [1, 2]. They account for 3–8% of all fractures [3, 4]. Pelvic injuries primarily occur in two separate age groups. The younger group mainly consists of men aged between 20 and 40 years. The predominant mechanisms of injury are traffic accidents, followed by other forms of high impact trauma [4–9]. The second peak mainly affects women around the aged of 80 years, typically due to low impact trauma, such as falls from a low height [4, 7].

Pelvic fractures are often associated with concomitant injuries and hemorrhage that can lead to hemodynamic instability [10, 11]. Mechanical instability of the dorsally disrupted pelvic ring as well as the low tissue pressure inside the pelvis leads to a simultaneous increase in intrapelvic volume and decrease in intrapelvic pressure. Consequently, the retroperitoneum can contain large amounts of blood before a tamponading effect is generated [12–14]. A study of Eastridge et al. shows that active pelvic hemorrhage occurs in 59% of patients with mechanically unstable pelvic fractures [15]. A retrospective analysis of the German Pelvic Trauma Registry shows that deaths of patients with pelvic fractures were mainly due to hemorrhage, while the pelvis was the predominant location of massive bleeding [16]. Balogh et al. describe hemorrhage as being the main cause of death following pelvic injury in both age groups [7].

Modern trauma guidelines and resuscitation protocols suggest, that pelvic binders should be used for hemorrhage control in the preclinical management of patients in case of mechanical instability of the pelvic ring and hemodynamic instability or if intrapelvic bleeding is suspected [17, 18]. Besides wrapping the pelvis with a sheet, commercially available circumferential pelvic binders are currently the only method for preclinical stabilization of the pelvis.

The application of a pelvic binder induces a circumferential pressure on the pelvis [19]. This leads to mechanical stabilization of the pelvic ring, reduction of fracture fragments, decrease in intrapelvic volume and increase in intrapelvic pressure [20–25]. Furthermore pelvic stabilization results in the reduction of bleeding from cancellous bone as well as diminished danger of further vascular and soft tissue injury by sharp bone fragments [20–25]. Hsu et al. showed that early application of pelvic binders significantly (*p* = 0.009) reduced transfusion requirements in patients with pelvic fractures. Furthermore, the mortality rate was lower when a pelvic binder was used, although statistical significance was not reached [24].

Placement at the level of the greater trochanter is recommended. Initial evaluation of the right application level can however be difficult in a preclinical setting. As a result, pelvic binders are regularly placed incorrectly, mostly higher than recommended, typically at the level of the iliac wing [25, 26].

At present, there are several different commercial models available. In Germany the SAM Pelvic Sling II (SAM Medical^®^, Tualatin, USA) and the T-POD (Trauma Pelvic Orthotic Device™, Pyng Medical, Richmond, Canada) are the ones most commonly used. They are both made of tightly woven cloth but differ from one another other regarding their width, shape and fastening mechanism. Currently there is no clear recommendation as to which model should be preferably used [27].

In our experience, preclinical personnel often report that the fastening mechanism of the T-POD appears to provide a more even distribution of pressure on the entire pelvis compared to the SAM Pelvic Sling II. This feature of the T-POD could prove beneficial in the initial treatment of pelvic fractures, as it could help reduce and stabilize pelvic fractures. The primary aim of this study was to compare the two models regarding their effectiveness in reducing the intrapelvic volume and increasing the intrapelvic pressure as well as the pressure in the pubic symphysis and sacroiliac joint when applied to cadavers with rotationally and vertically unstable pelvic fractures (AO/OTA classification 61-C1.1).

As correct placement of pelvic binders can be difficult in the prehospital phase, incorrect positioning is often first detected after arriving in the emergency room. In these cases, the T-POD appears to be partially positioned over the greater trochanter in most cases due to its greater width, whereas the SAM Pelvic Sling II is often placed entirely above or below the targeted anatomical structure. The second aim was to determine whether centering the two models at a level above or below the recommended level affects the parameters.

## Materials and methods

The experiments were conducted on five fresh-frozen human cadaveric specimens, one female and four males, aged between 56 and 94. In order to simulate the joint mobility and the tissue characteristics of living patients as realistic as possible, all experiments were carried out at room temperature. Other research groups used a similar experimental set-up with unfixed cadavers to study pelvic injuries and the effect of commercial pelvic binders [20, 21, 28–30]. All measurements were conducted on two consecutive days.

### Surgical approach: generating a type C pelvic injury

**Fig. 1:**
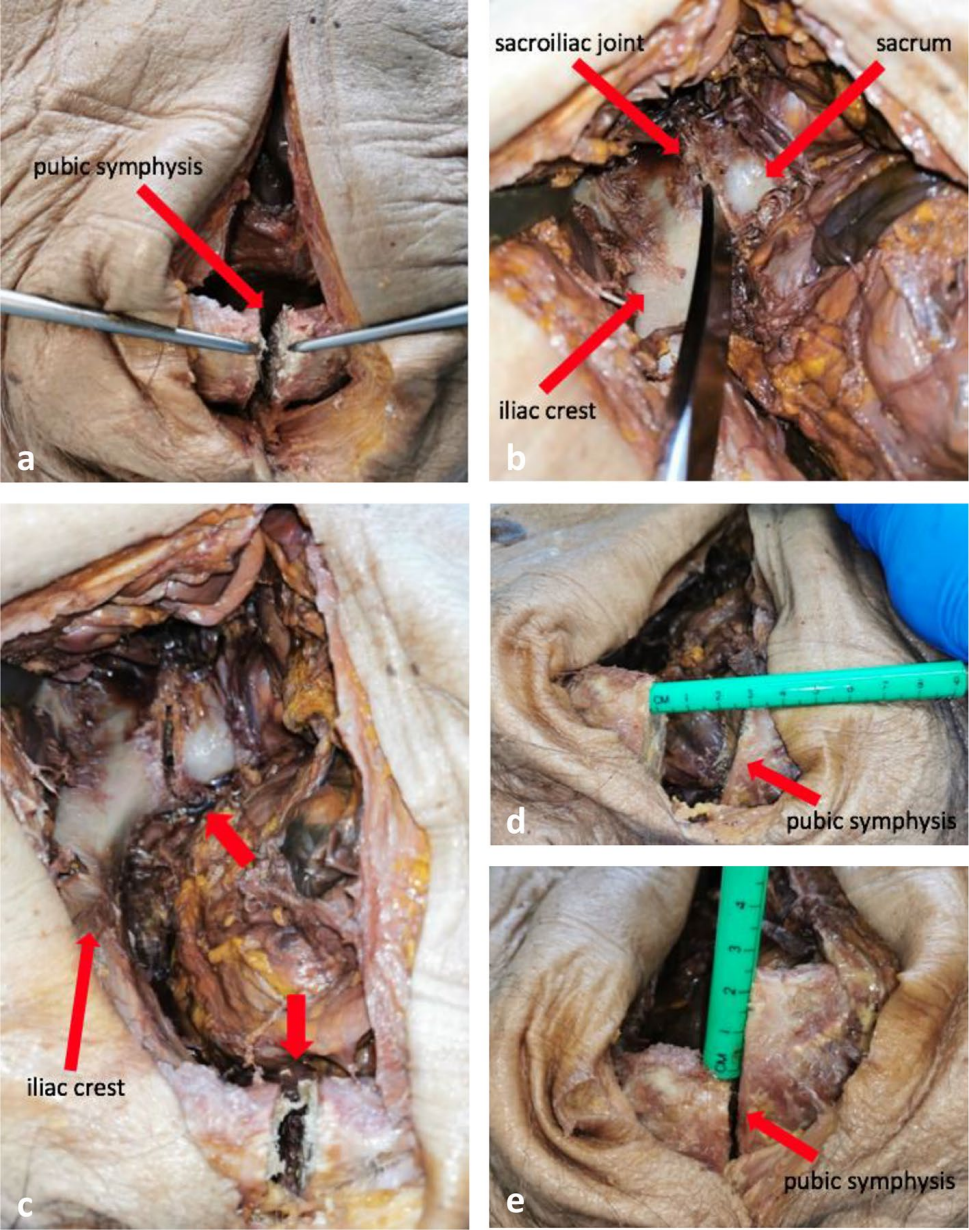
(**a**) Dissection of the pubic symphysis, (**b**) dissection of the sacroiliac joint, (**c**) diastasis of the pubic symphysis and the sacroiliac joint (red arrows), (**d**) clinical testing for horizontal instability, (**e**) clinical testing for vertical instability

During inspection and clinical examination there were no signs of previous trauma to the pelvis. In the initial phase rotationally and vertically unstable pelvic fractures (AO/OTA classification 61-C1.1) were produced in all human specimens. This type of fracture is characterized by a complete disruption of both the pubic symphysis and the posterior arch. Dissection of the corpses therefore included transection of the pubic symphysis and one sacroiliac joint. Both structures were reached through an anterior approach. A medial skin incision of approximately 10–15 cm was made caudal to the umbilicus. When reaching the pubic symphysis, diastasis of the joint was created by cutting the superior and inferior pubic ligaments and resecting the interpubic disc (Fig. 1a). Access to the lower abdomen and pelvis was gained by an incision of the linea alba proximal to the pubic symphysis. The sacroiliac joint was then reached by mobilising the intraabdominal and pelvic viscera. Injuries of internal organs, especially the bladder and bowel, were avoided in all cases. The superior pubic ramus served as a guide structure for the localisation of the sacroiliac joint. After incision of the anterior joint capsule and mobilisation of the iliopsoas muscle a chisel was used to disrupt the sacroiliac joint. Anatomical structures disrupted by this maneuver included the anterior, interosseous and posterior sacroiliac ligaments as well as adjacent fragments of the ilium and sacrum (Fig. 1b-c). The iliolumbar ligament was cut at its insertion at the iliac crest. Distally the sacrotuberous and the sacrospinous ligaments were disrupted at the ischial tuberosity and the ischial spine. Subsequently the pelvis were examined for the presence of mechanical instability by means of manual compression. Clinical examination included craniocaudal mobilization of the hemipelvis as well as its internal and external rotation to check for both vertical and rotational instability (Fig. 1d-e). After fulfilling these criteria reproduction of type C pelvic ring fractures (AO/OTA) was considered successful. During the dissection, no previous injuries to the bony pelvis as well as adjacent organ systems and soft tissues could be detected in any of the cadavers. Each human specimen was dissected immediately before carrying out the series of experiments.

### Experimental workflow/setup and parameters

**Fig. 2:**
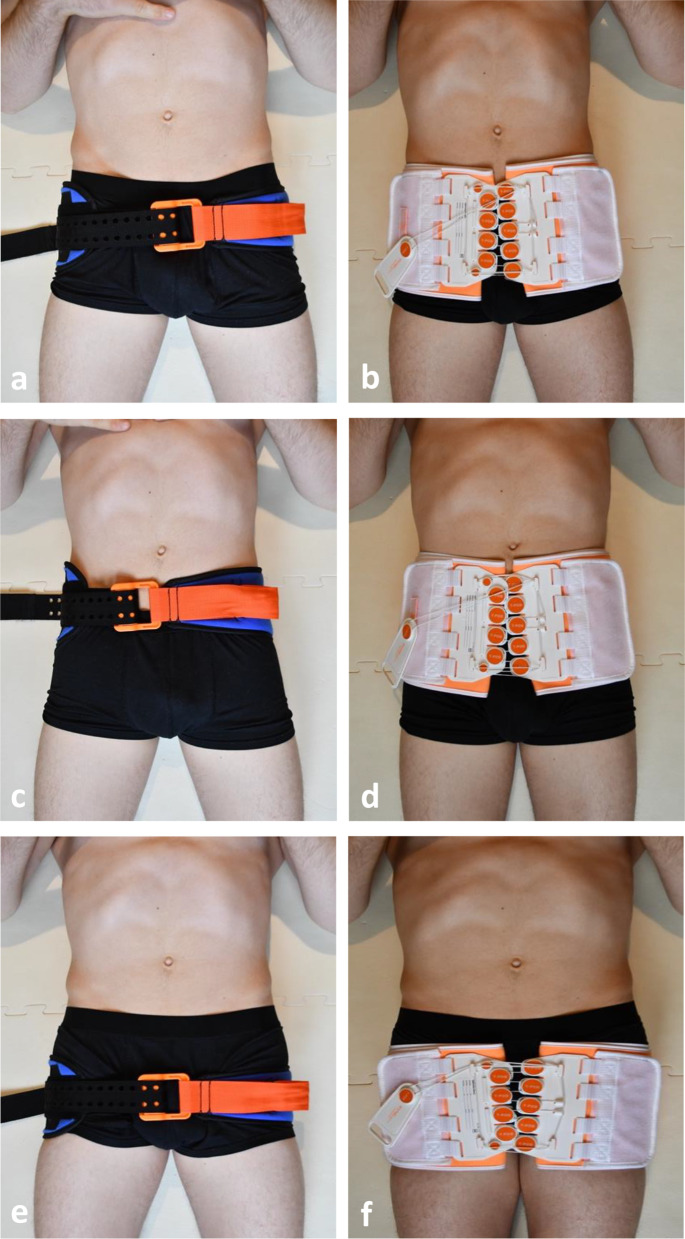
Application of the two pelvic binder models at three different levels: (**a**) SAM Pelvic Sling II at the greater trochanter, (**b**) T-POD at the greater trochanter, (**c**) SAM Pelvic Sling II at the iliac crest, (**d**) T-POD at the iliac crest, (**e**) SAM Pelvic Sling II below the greater trochanter, (**f**) T-POD below the greater trochanter

In the first part of our study, we measured several parameters and their changes before and during the use of a T-POD and a SAM Pelvic Sling II on the cadavers in three different positions. Initially baseline measurements of the intrapelvic volume were taken, before the first pelvic binder was applied. Furthermore, baseline values of the vesical pressure, the pressure inside the pubic symphysis and the sacroiliac joint were determined before applying the pelvic binders at each level. The legs were then rotated inwards and tape was attached slightly above the knees, as suggested by Gardner et al. [31]. The pelvic binders were applied in the same order throughout the test series. The experiments were first conducted using the SAM Pelvic Sling II and then the T-POD. Each pelvic binder was centered and fastened at three different levels (Fig. 2). These were defined as follows:

1. Recommended level of application: Greater trochanter.
2. High level of application: Iliac crest.
3. Low level of application: Below the greater trochanter.

Before and during the application of SAM Pelvic Sling II and T-POD in these positions, the following parameters were measured.

#### Vesical pressure

The vesical pressure served as a correlate for the intrapelvic pressure. A urinary catheter with a pressure sensor was placed inside the bladder. The emptied bladder was then filled using 25 ml of 0.9% sodium chloride solution. This method is currently used as standard, especially in intensive care, to monitor the pressure in the abdominal cavity [32]. The vesical pressure was measured in cmH_2_O.

#### Pressure inside the pubic symphysis and the sacroiliac joint

Compression pressures within the pubic symphysis and the sacroiliac joint before and during application of the pelvic binders were measured using digital force gauges. These were inserted in the respective joints during the dissection of the cadavers. The model we used had a diameter of 16.5 mm and a width of 6.8 mm. According to the manufacturer, these gauge devices measure forces up to 1000 N with an accuracy of ±0.5% [33].

#### Intrapelvic volume

The intrapelvic volumes were calculated on the basis of pelvic CT-scans which were obtained both before and during application of the pelvic binders. The slice thickness was 1.0 mm. The collected data sets were then viewed and analysed using the DICOM viewer Horos for Mac OS X (version: Horos v3.3.5). Initially, the images were examined for radiological signs of fresh and old injuries to the bony pelvis and the adjacent soft tissues. In one cadaver a total hip endoprothesis of the right hip joint was found. This was not considered an exclusion criterion. After the initial assessment of the CT images, the measurements of the pelvic volumes were carried out using Horos for Mac OS X as well. Currently, there is no standard radiological method for determining the intrapelvic volume. We therefore used an approach introduced by Kaufmann et al. In their study involving 142 patients, they presented a method based on computer tomography. The procedure uses ROIs (regions of interest) which are manually annotated into each CT-slice of a data set. Given the thickness of the slices and the area of the ROIs, the volume can then be determined using a DICOM viewer [34].

**Fig. 3:**
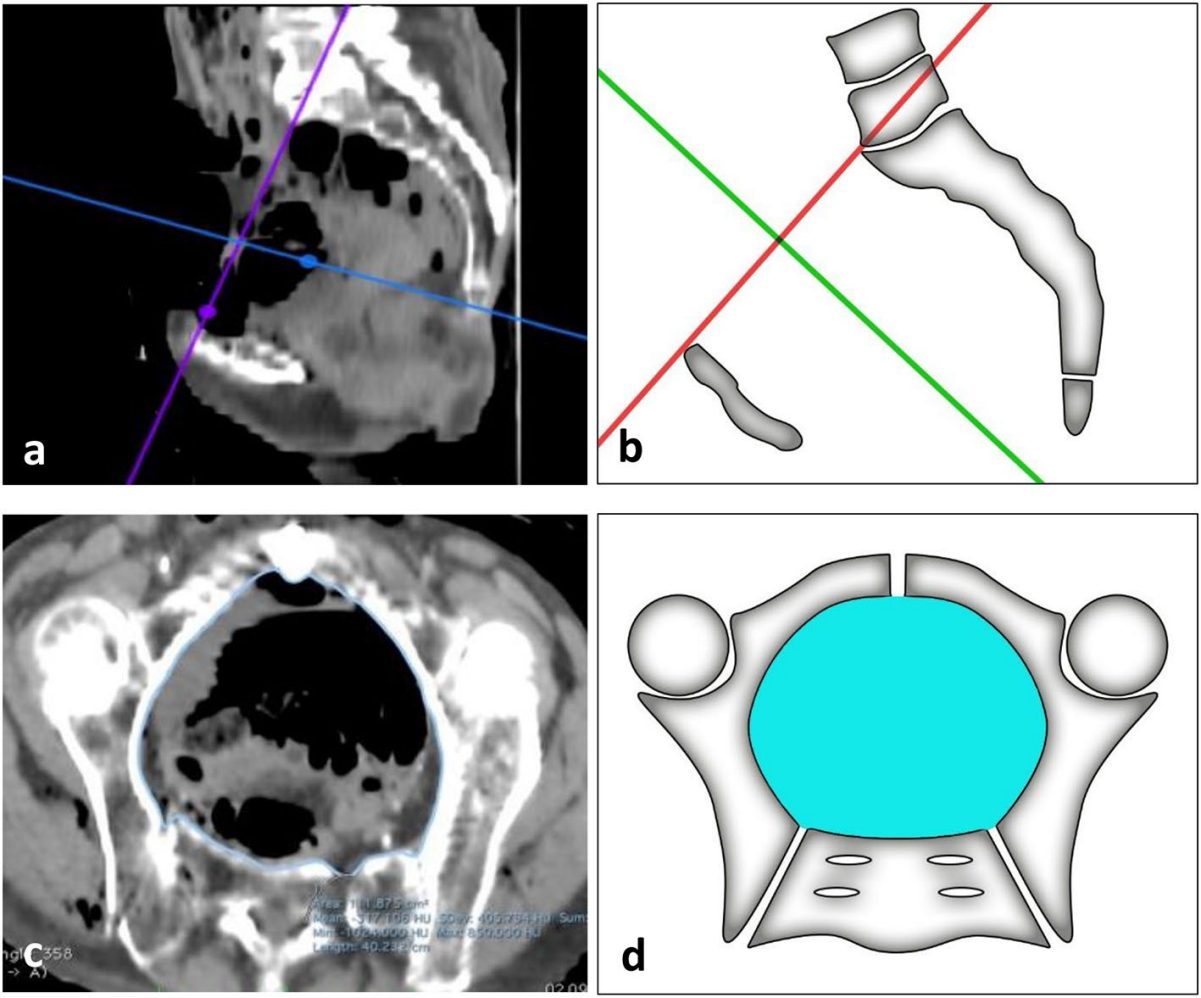
(**a**) Tilting of the pelvis to visualise and define the terminal line using Horos for Mac OS X with (**b**) the corresponding sketch and (**c**) manual segmentation of ROIs in the pelvis with (**d**) the associated sketch

Following this example, we defined the pelvic inlet along the terminal line as the upper limit of the inner pelvic volume (Fig. 3a). The lower limit was set at the level of the ischial tuberosities. ROIs were then manually inserted in each parallel slice between these two boundaries. The ROIs outlined the inner pelvic area along the osseous pelvis (Fig. 3b). Most soft tissues such as muscles were included in these regions. In planes without a closed osseous ring, defined anatomical structures were used as outer borders in addition to the bone. The areas of the ROIs were automatically determined by Horos for Mac OS X in cm^2^. With a constant slice thickness of 1.0 mm the volume was then calculated and given in cm^3^.

#### Statistical analysis

Following these measurements, the values obtained were used to calculate differences in pressure and volume before and after the application of the respective pelvic binder. The results were then tested for statistical significance using a paired t-test, whereby the significance level was defined as *p* < 0.05. The paired t-test is the optimal test as it calculates with the original values. Since the precondition for the paired t-test is the normal distribution of the differences and this was not always given, the Wilcoxon signed-rank test was calculated in these cases in order to better estimate the probability of error. The calculations were used to compare the two pelvic binders as well as the three levels of application. SAS (9.4) was used for this statistical analysis.

## Results

### Vesical pressure

**Fig. 4:**
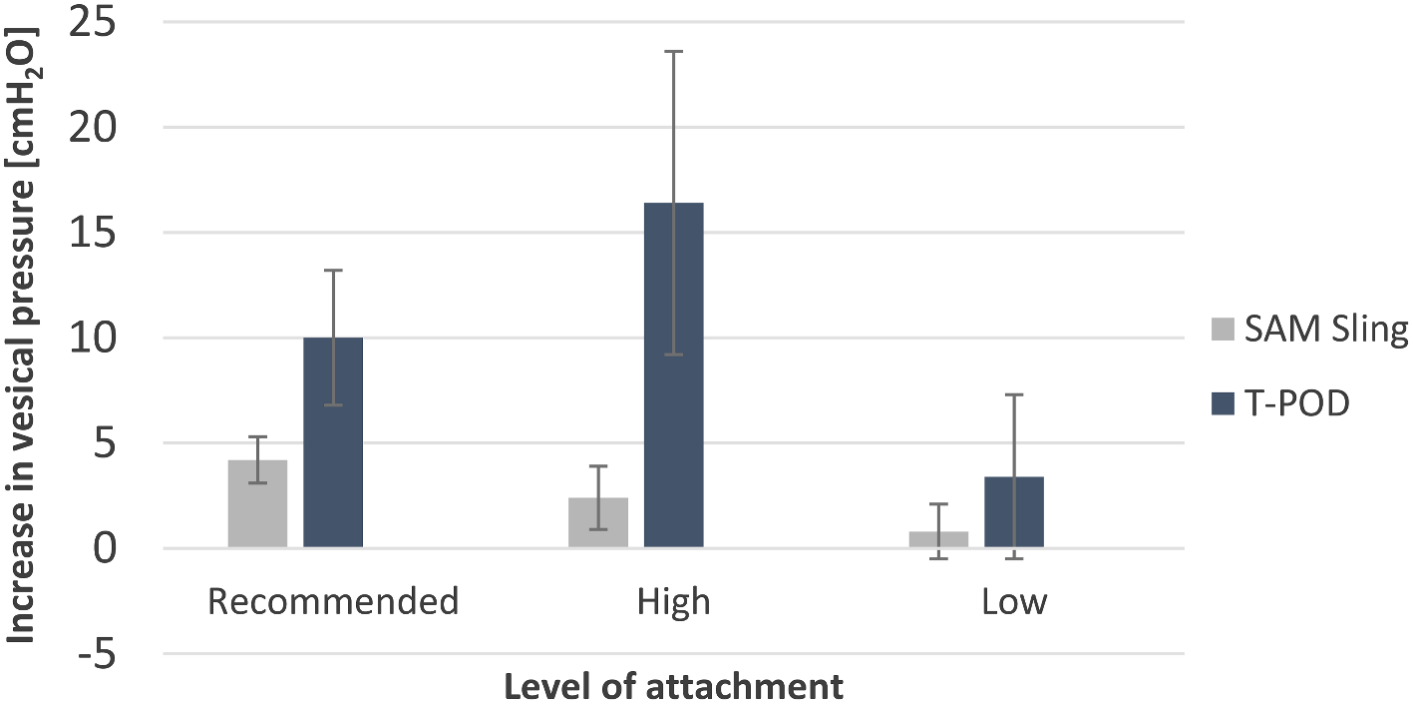
Mean increase in vesical pressure resulting from the application of a SAM Pelvic Sling II and a T-POD at three different levels (Recommended: greater trochanter, High: iliac crest, Low: below the greater trochanter)

At the recommended level (greater trochanter), application of both pelvic binders led to an increase in vesical pressure (Table 1). However, the result was only significant for the T-POD (10.0 ± 3.2 cmH_2_O, *p* = 0.002) (Fig. 4).

**Table 1:** Mean vesical pressure (in cmH_2_O) before and after application of a SAM Pelvic Sling II and a T-POD at three different levels (greater trochanter, iliac crest, below the greater trochanter)

Level of application	Greater trochanter	Iliac crest	Below greater trochanter
**SAM Pelvic Sling II**			
Before application	9.6	10.4	10.4
After application	13.8	12.8	11.2
**T-POD**			
Before application	10.4	10.2	10.6
After application	20.4	26.6	14.0

When applied at a level below the greater trochanter, none of the pelvic binders achieved a statistically significant increase in vesical pressure (Fig. 4).

Attachment of the T-POD and SAM Pelvic Sling II at the level of the iliac crest resulted in a significant increase in vesical pressure in both cases (16.4 ± 7.2 cmH_2_O, *p* = 0.007 and 2.4 ± 1.5 cmH_2_O, *p* = 0.024 respectively) (Fig. 4).

Comparing the two pelvic binders, the pressure changes achieved with the T-POD were significantly higher than those of the SAM Pelvic Sling II at both the level of the greater trochanter (5.80 ± 3.27 cmH_2_O, *p* = 0.017) and the level of the iliac crest (14.00 ± 8.57 cmH_2_O, *p* = 0.022). The comparison of the pelvic binders applied below the greater trochanter showed no significant difference of pressure increase (2.60 ± 4.51 cmH_2_O, *p* = 0.267).

### Intrapelvic volume

**Fig. 5:**
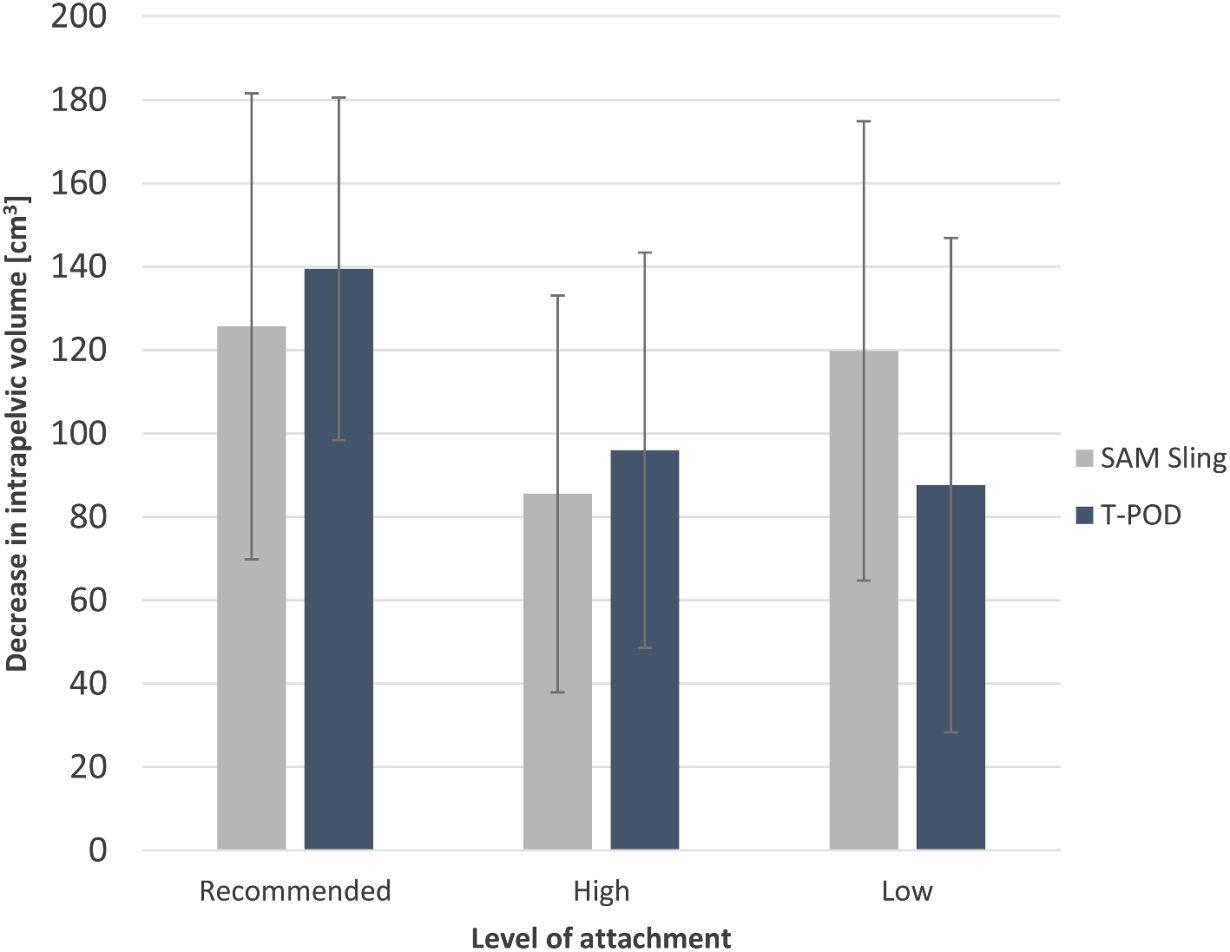
Mean decrease in intrapelvic volume resulting from the application of a SAM Pelvic Sling II and a T-POD at three different levels (Recommended: greater trochanter, High: iliac crest, Low: below the greater trochanter)

Application of both the SAM Pelvic Sling II and T-POD at all three levels led to a reduction in intrapelvic volume (Table 2). With one exception, the use of both pelvic binder models resulted in a significant volume reduction when applied at the different positions. Only the placement of the T-POD below the greater trochanter did not lead to statistical significance (87.55 ± 59.28 cm^3^, *p* = 0.060) (Fig. 5).

**Table 2:** Mean intrapelvic volume (in cm^3^) before and after application of a SAM Pelvic Sling II and a T-POD at three different levels (greater trochanter, iliac crest, below the greater trochanter)

Level of application	Greater trochanter	Iliac crest	Below greater trochanter
**SAM Pelvic Sling II**			
Before application	1275	1275	1275
After application	1150	1190	1156
**T-POD**			
Before application	1275	1275	1275
After application	1136	1179	1188

The direct comparison of the two binders showed that there was no significant difference in volume reduction when positioned at the trochanteric level (13.85 ± 31.37 cm^3^, *p* = 0.442).

When applied below the trochanters, the SAM Pelvic Sling II provides a significantly greater volume reduction than the T-POD (-32.26 ± 7.52 cm^3^, *p* = 0.003). On the other hand, when placed at the iliac crest, the T-POD achieved a significantly greater decrease in intrapelvic volume compared to the SAM Pelvic Sling II (10.45 ± 5.45 cm^3^, *p* = 0.031).

### Pressure in the pubic symphysis and the sacroiliac joint

Placement of the two pelvic binders at the different levels led to a pressure increase both in the pubic symphysis (Table 3) and the sacroiliac joint (Table 4).

**Table 3:** Mean pressure in the pubic symphysis (in N) before and after application of a SAM Pelvic Sling II and a T-POD at three different levels (greater trochanter, iliac crest, below the greater trochanter)

Level of application	Greater trochanter	Iliac crest	Below greater trochanter
**SAM Pelvic Sling II**			
Before application	0.20	0.20	0.10
After application	1.60	0.54	0.66
**T-POD**			
Before application	0.18	0.16	0.12
After application	2.10	1.22	1.42

**Table 4:** Mean pressure in the sacroiliac joint (in N) before and after application of a SAM Pelvic Sling II and a T-POD at three different levels (greater trochanter, iliac crest, below the greater trochanter)

Level of application	Greater trochanter	Iliac crest	Below greater trochanter
**SAM Pelvic Sling II**			
Before application	0.52	0.30	0.58
After application	2.63	1.83	3.70
**T-POD**			
Before application	0.30	0.23	0.30
After application	2.38	1.75	1.98

**Fig. 6:**
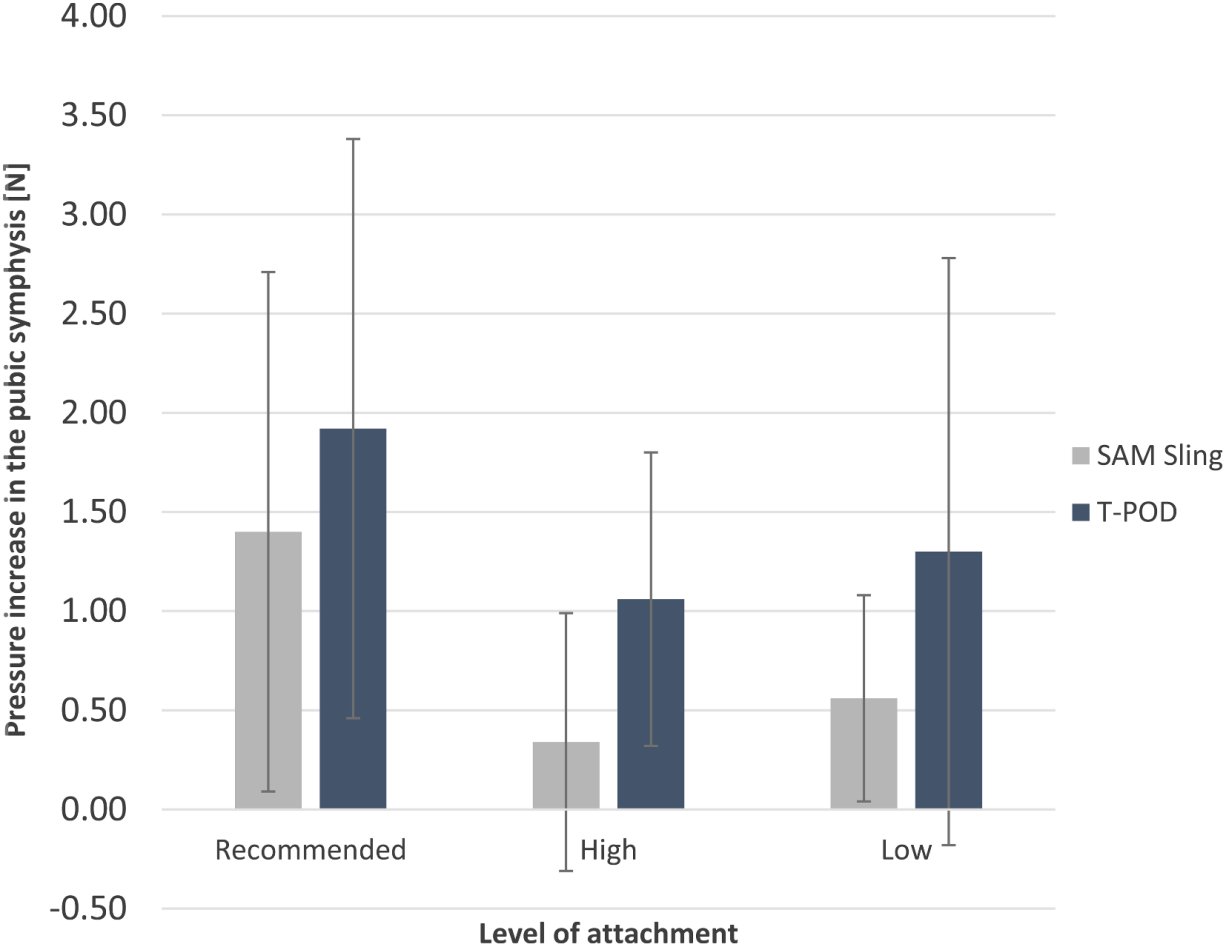
Mean increase in pressure in the pubic symphysis resulting from the application of a SAM Pelvic Sling II and a T-POD at three different levels (Recommended: greater trochanter, High: iliac crest, Low: below the greater trochanter)

Application of the T-POD at the trochanteric level and the iliac crest resulted in a statistically significant increase in pressure in the pubic symphysis (1.92 ± 1.46 N, *p* = 0.043 and 1.06 ± 0.74 N, *p* = 0.033 respectively) (Fig. 6). Application of the SAM Pelvic Sling II at the three different levels did not lead to a significant pressure increase in the pubic symphysis (Fig. 6).

**Fig. 7:**
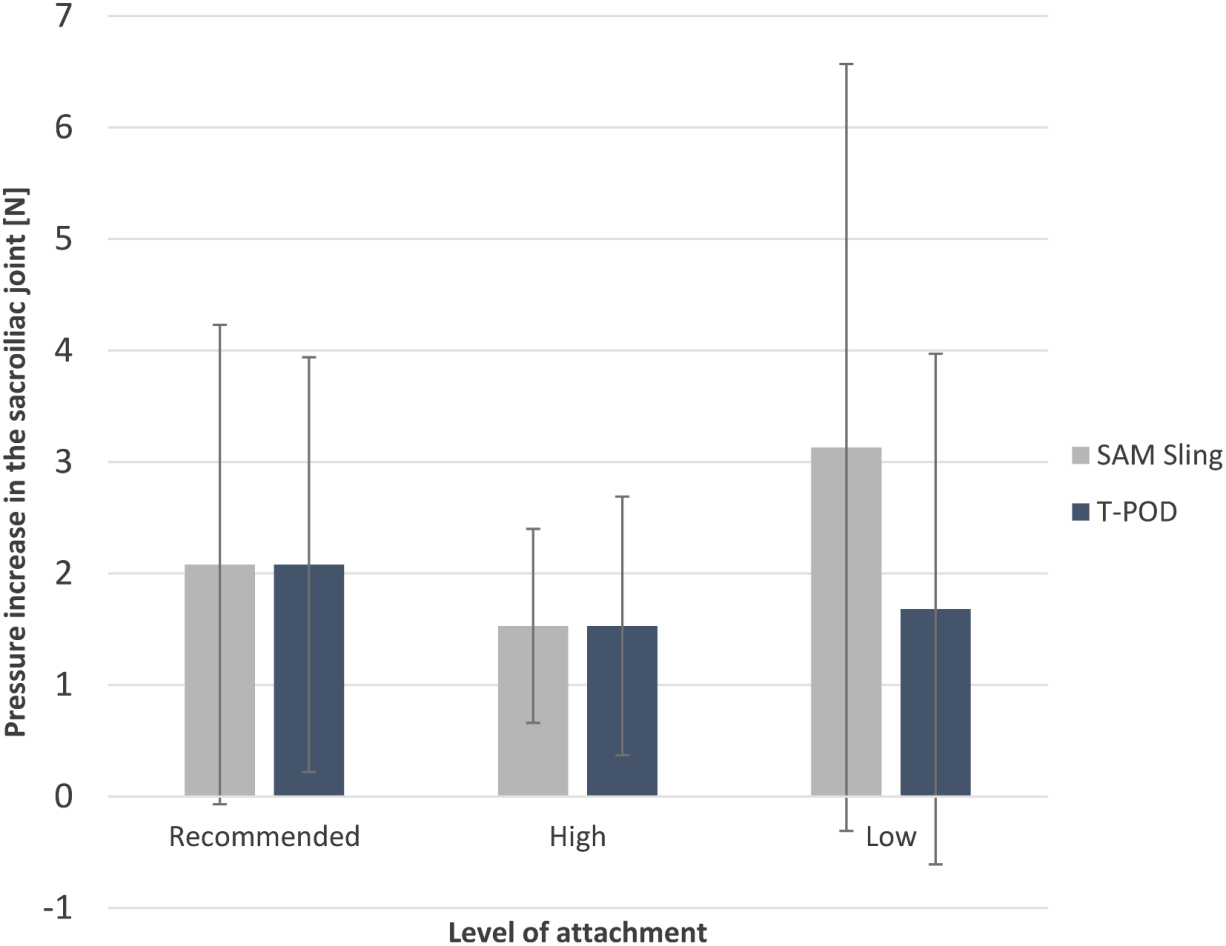
Mean increase in pressure in the sacroiliac joint resulting from the application of a SAM Pelvic Sling II and a T-POD at three different levels (Recommended: greater trochanter, High: iliac crest, Low: below the greater trochanter)

Regarding the sacroiliac joint, only the SAM Pelvic Sling II placed at the iliac crest significantly increased the pressure (1.53 ± 0.87 N, *p* = 0.039) (Fig. 7).

### T-POD – comparison of different levels of application

When comparing the application at the recommended level (greater trochanter) with both of the other application levels, there were no significant differences in intrapelvic pressure increase or volume reduction (Table 5).

**Table 5:** T-POD – comparison of different levels of application (Recommended: greater trochanter, High: iliac crest, Low: below the greater trochanter)

T-POD				
	Increase in vesical pressure [cmH_2_O]		Decrease in intrapelvic volume [cm³]	
	Recommended vs. High	Recommended vs. Low	Recommended vs. High	Recommended vs. Low
Mean	-6.40	6.60	43.55	51.97
Standard deviation	5.73	2.19	33.30	38.66
p-value	0.067	0.063	0.079	0.075

### SAM pelvic Sling II – comparison of different levels of application

Positioning the SAM Pelvic Sling II below the greater trochanter led to a significantly lower increase in vesical pressure than the attachment at the recommended level (greater trochanter) (3.40 ± 1.52 cmH_2_O, *p* = 0.007) (Table 6). Fastening the binder at the level of the iliac crest resulted in a significantly smaller reduction of the intrapelvic volume compared to the recommended positioning (greater trochanter) (40.15 ± 14.57 cm^3^, *p* = 0.012) (Table 6).

**Table 6:** SAM Pelvic Sling II- comparison of different levels of application (Recommended: greater trochanter, High: iliac crest, Low: below the greater trochanter)

SAM Pelvic Sling II				
	Increase in vesical pressure [cmH_2_O]		Decrease in intrapelvic volume [cm³]	
	Recommended vs. High	Recommended vs. Low	Recommended vs. High	Recommended vs. Low
Mean	1.80	3.40	40.15	5.85
Standard deviation	1.92	1.52	14.57	8.14
p-value	0.105	0.007	0.012	0.246

## Discussion

### Recommended level of application (greater trochanter)

When applied at the level of the greater trochanter, the T-POD achieved a significant decrease in intrapelvic volume and a significant increase in vesical pressure. Positioning of the SAM Pelvic Sling II as recommended led to a significant reduction in intrapelvic volume as well, but no statistical significance was achieved regarding the increase in vesical pressure. Furthermore, only the T-POD achieved a significant pressure increase in the pubic symphysis when applied at the trochanteric level.

Two studies show a significant reduction of pubic symphysis diastasis in patients with unstable pelvic fractures after proper application of the T-POD [21, 35]. As the width of the symphyseal diastasis correlates with intrapelvic volume, the results of these two studies are comparable to the significant reduction in intrapelvic volume we achieved when applying the T-POD as recommended [36].

In a study similar to ours, Morris et al. measured the intrapelvic pressure in six unembalmed human cadaveric specimens with surgically created unstable pelvic injuries (C61-C1 OA/OTA) when applying a T-POD at the level of the greater trochanters and bandaging the lower limbs [22]. To record the pressure inside the pelvis a balloon was placed in the retropubic space. Application of the T-POD resulted in a significant increase in intrapelvic pressure leading to a pressure of 24 cmH_2_O (SE = 5) (*p* < 0.036). Additional bandaging of the lower limbs led to a pressure of 31 cmH_2_O (SE = 7). In our study, the mean pelvic pressure obtained by combining a T-POD over the greater trochanter and lower limb bandaging was lower than the results described by Morris et al., which might be due to a pressure-induced leakage of fluid from the bladder into the ureters [22]. In contrast to our study, the effects of pelvic binder application at a level above and below the greater trochanters were not investigated by Morris et al.

In summary, application of the T-POD at the recommended level produces significant results in terms of increasing vesical pressure, reducing intrapelvic volume and increasing pressure in the pubic symphysis, while the SAM Pelvic Sling II achieved a significant reduction in intrapelvic volume when placed at the level of the greater trochanter.

### Application above and below the greater trochanter

When applied at the iliac crest, both the T-POD and the SAM Pelvic Sling II achieved a significant increase in vesical pressure. When positioned below the greater trochanter, none of the pelvic binders reached a statistically significant intrapelvic pressure increase.

Placement of the T-POD at the iliac crest resulted in a significant pressure increase in the pubic symphysis, while applying the SAM Pelvic Sling II at this level significantly increased the pressure in the sacroiliac joint.

Application of the T-POD and SAM Pelvic Sling II at the different levels led to a significant reduction in intrapelvic volume. An exception was the attachment of the T-POD below the greater trochanter, which did not lead to a statistically significant result.

When comparing the attachment at the recommended level with a high or low positioning, the T-POD showed no significant differences in volume reduction or vesical pressure increase.

With the SAM Pelvic Sling II the volume reduction at the level of the iliac crest was significantly lower than the recommended placement. Positioning below the greater trochanter, on the other hand, led to a significantly smaller increase in vesical pressure compared to the placement at the trochanteric level.

A study of Bonner et al. showed similar results in patients with open-book fractures in whom the use of a SAM Pelvic Sling II at a higher level than the greater trochanters led to a significantly greater (*p* < 0.01) remaining symphysial diastasis compared to the correct placement [26].

A retrospective analysis of 167 patients who had a SAM Pelvic Sling II applied showed that in 50% of the cases the pelvic binders had been placed correctly, 39% had been applied higher than recommended [26]. In a study of Bakhshayesh et al. in which 90% of patients received a T-POD, placement at the level of the greater trochanters was seen in 81% of the cases [37]. Comparing these two studies, prehospital application at the level of the greater trochanters seems to be achieved more readily with the T-POD than the SAM Pelvic Sling II. Bakhshayesh et al. suggest that this may be due to the larger width of the T-POD, which facilitates covering the greater trochanters [37].

According to the manufacturer, the closing mechanism of the T-POD leads to an even pressure on the pelvis under the entire pelvic binder [38]. Due to its width, parts of the pelvic binder may lie over and exert pressure on the greater trochanters in all three levels of application, resulting in a reduction in intrapelvic volume and an increase in intrapelvic pressure. This may be the reason, the T-POD showed no significant differences in volume reduction or pressure increase when comparing the incorrect levels of attachment with the recommended placement.

In a study very similar to ours, Bottlang et al. investigated the effect of a prototype pelvic strap on unstable type 61-C1 pelvic fractures (AO/OTA) [39]. The aim of this study was to evaluate the ideal level of application of pelvic binders. They used seven human cadaveric specimens, which they dissected via an anterior approach, as we did in our study. Bottlang et al. measured the application force needed to close the symphyseal diastasis and investigated the effect on intraperitoneal pressure when applying a pelvic binder at different levels. These included, as in our study, application at the level of the greater trochanters and the iliac crest, however, a position lower than the greater trochanters was not investigated. In contrast to their study, we measured vesical pressure as a correlate for intrapelvic pressure, as bleeding sites are often located in the retroperitoneum. The lowest application force required by Bottlang et al. for a complete reduction of the pubic symphysis was needed when applying the pelvic binder at the level of the greater trochanters. When positioning the pelvic binder at this level, the intraperitoneal pressure increased by 6.2 ± 5.8 mmHg. Our results for increase in vesical pressure when attaching the T-POD and the SAM Pelvic Sling II at the recommended level are within that range. Like Bottlang et al., we also achieved a greater pressure increase when applying the T-POD at the level of the iliac crest compared to the recommended level. Again, our results lie within their range. The greater increase in pressure achieved with a high level of application compared to attachment at the recommended level may be due to a greater force exerted on the lower abdomen and the bowel when a pelvic binder is applied over an area not completely surrounded by the pelvic ring. On the one hand, this could mean that a high application could be beneficial for the intrapelvic pressure increase and the resulting tamponading effect. On the other hand, measurement of intraperitoneal pressure or vesical pressure may not be appropriate to evaluate the effect of pelvic binders on the intrapelvic pressure.

In another cadaveric study, Prasarn et al. investigated the motion of unstable pelvic fractures when applying a T-POD over the greater trochanter or the iliac crest [40]. Their results show, that placement of the pelvic binder at the recommended site leads to significantly less (*p* < 0.05) motion of the fracture compared to the higher level of application.

In summary, the ideal level of application for both the T-POD and the SAM Pelvic Sling II is at the level of the greater trochanters. This is particularly important for the SAM Pelvic Sling II as deviations from the recommended attachment level led to significant differences in intrapelvic volume reduction and pressure increase. The T-POD was less susceptible to incorrect positioning showing no significant difference in intrapelvic volume reduction and pressure increase when placed in a position other than recommended. It even achieved a greater increase in intraperitoneal and vesical pressure when applied at the iliac crest compared to the recommended level [39]. Nevertheless, the T-POD shows better results in terms of fracture reduction and stabilization when placed correctly [39, 40]. A major advantage of the T-POD over the SAM Pelvic Sling II lies in its greater width, which facilitates prehospital application at the level of the grater trochanters [26, 37]. In particular, placement at a level below the greater trochanter appeared to be disadvantageous in our study, as neither pelvic binder achieved a statistically significant increase in vesical pressure and application of the T-POD did not lead to a significant reduction in intrapelvic volume when applied in this position.

### Direct comparison of T-POD and SAM pelvic Sling II

When comparing the two pelvic binders positioned at the recommended level, there was no significant difference in volume reduction, however, increase in vesical pressure was significantly higher with the T-POD.

When applied at the iliac crest, the T-POD showed a significantly greater increase in vesical pressure and a significantly greater reduction in intrapelvic volume than the SAM Pelvic Sling II. On the other hand, the SAM Pelvic Sling II led to a significantly greater volume reduction at the application level below the greater trochanter compared to the T-POD.

The significantly higher vesical pressure achieved with the T-POD compared to the SAM Pelvic Sling II at both the recommended level of application and the iliac crest, as well as the lack of statistical significance of the pressure increase when applying the SAM Pelvic Sling II as recommended, could again be due to the different width of their straps and the different closing mechanisms.

In a cadaveric study by Knops et al. on 16 embalmed specimens, three different pelvic binders (Pelvic Binder, T-POD and SAM Pelvic Sling II) were compared in regard to the reduction of pelvic fractures (Tile A-C) when applied as recommended [23]. Each pelvic binder achieved a complete reduction of the pubic diastasis, however, the mean pulling force required was 68 ± 10 N lower with the T-POD than with the SAM Pelvic Sling II (*p* < 0.01).

In summary, both the T-POD and the SAM Pelvic Sling II showed a significant reduction in intrapelvic volume. The effect, however, was greatest with the T-POD when applied at the level of the greater trochanter and the iliac crest. The T-POD also demonstrated better results with regard to intravesical pressure increase than the SAM Pelvic Sling II when positioned at these levels. However, when applied below the greater trochanter, the SAM Pelvic Sling II achieved a significantly greater intrapelvic volume reduction.

### Limitations

One limiting factor of our study is the small sample size, which may have contributed to a lack of statistical significance in some of our findings. However, there have been several comparable studies in the past with a similar number of human cadaveric specimens [22, 30, 36, 39, 40].

Another limiting factor could be the use of the vesical pressure as a correlate for the intrapelvic pressure. Morris et al. suggest, that there may be a pressure-related leakage of fluid from the bladder into the ureters [22]. As a result, the pressure measured in the bladder may not fully reflect the intrapelvic pressure. Furthermore, in some cases the increase in vesical pressure may have been due to a direct force exerted on the bladder via the lower abdomen rather than an actual increase in intrapelvic pressure. Nevertheless, we believe that our results can be used to compare the two pelvic binders. Overall, this method was used to avoid further manipulation of the cadavers and to ensure that the characteristics of our experimental setup were as realistic as possible.

Dissection of the cadavers was performed by the same orthopedic surgeon in all cases. Although the fracture patterns created may differ from injuries caused by actual trauma, this approach was necessary to ensure standardization of our experiments. In addition, we investigated the effect of pelvic binders on only one type of fracture (AO/OTA classification 61-C1.1). Further studies are needed to examine the effectiveness of the T-POD and SAM Pelvic Sling II on different types of pelvic injuries.

We used non-embalmed fresh human cadaveric specimens to simulate realistic tissue characteristics. Our results must nevertheless be viewed critically, as this is an experimental study and our model may not fully represent an injured patient following trauma. Future clinical studies on this topic are therefore necessary in order provide definitive recommendations.

## Conclusion

In our study both the T-POD and the SAM Pelvic Sling II showed an increase in vesical pressure and pressure in the pubic symphysis as well as a reduction in intrapelvic volume when applied as recommended. However, only the T-POD achieved significant results for all three parameters. Furthermore, the T-POD was less susceptible to incorrect positioning showing no significant difference in intrapelvic pressure increase and volume reduction when applied in positions above or below the greater trochanter compared to the recommended level of application. We therefore recommend that the T-POD should be used as the preferred device for prehospital emergency pelvic stabilisation. It must be noted, however, that the T-POD did not achieve statistically significant results in intrapelvic pressure increase and volume reduction when applied below the greater trochanters. This level of application, therefore, appears to be particularly disadvantageous and should be strictly avoided when using a T-POD.

## Data Availability

All data and statistics are stored in the archive of the BG Klinik Ludwigshafen and are available on request from the corresponding author.
