# Correction: Comparison of T-POD and SAM Pelvic Sling II and the influence of attachment level in the initial management of unstable pelvic type C injuries – a cadaveric study

**DOI:** 10.1186/s12245-025-00939-8

**Published:** 2025-07-02

**Authors:** Maxim Privalov, Malte Junge, Matthias Karl Jung, Sven Yves Vetter, Jochen Franke, Svetlana Hetjens, Paul Alfred Grützner, Holger Stadthalter, Niko R.E Schneider

**Affiliations:** 1 Department for Trauma and Orthopaedic Surgery, BG Klinik Ludwigshafen, Ludwig- Guttmann-Str. 13, Ludwigshafen, 67071 Germany; 2 Department for Trauma and Orthopaedic Surgery, Tauernklinikum, Paracelsusstraße 8, Zell am See, 5700 Austria; 3 Department of Medical Statistics, Biomathematics and Data Processing, Universitätsklinikum Mannheim, Theodor-Kutzer-Ufer 1-3, Mannheim, 68167 Germany; 4 Friedrich-Alexander-University Erlangen-Nürnberg, Krankenhaus-Str. 12, Erlangen, 91054 Germany; 5 Department for Trauma and Orthopaedic Surgery, AUVA Unfallkrankenhaus, Dr.- Franz-Rehrl-Platz 5, Salzburg, 5010 Austria; 6 Department of Anesthesiology, Operative Intensive Care Medicine and Pain Therapy, University Hospital of Gießen and Marburg, Gießen, Germany; 7 Medical Faculty Heidelberg, Department of Anesthesiology, Heidelberg University, Heidelberg, Germany

**Correction: Int J Emerg Med 17**,** 34 (2024)**

10.1186/s12245-024-00610-8

Following publication of this article, it has been brought to our attention that the name of a contributing author, Dr. Niko R.E. Schneider, was inadvertently omitted from the author’s list due to a miscommunication among the co-authors.

The correct authorship is provided in this erratum.

As a result of this correction, Dr. Niko R.E Schneider now serves as the corresponding author, replacing Dr. Holger Stadthalter.

The updated Acknowledgements and Author’s contributions statement can be found below:

**Acknowledgements**

**Incorrect**

We would like to thank Dr. Svetlana Hetjens from the department of Medical Statistics, Biomathematics and Information Processing for her contribution to the statistical data analysis of this study.

**Correct**

We would like to thank Dr. Svetlana Hetjens from the department of Medical.

Statistics, Biomathematics and Information Processing for her contribution to.

the statistical data analysis of this study.

**We would also like to thank Dr sc. hum. Sara Doll from University of Heidelberg**,** Institute of Anatomy and Cell Biology**,** Prosecture & Anatomical Collection for providing and assisting with the preparation of the cadavers.**

**Author’s contributions**

**Incorrect**

Conceptualisation: H.S. Data curation: M.J., M.P. Formal analysis: M.J., S.H. Investigation: H.S., M.J. Methodology: H.S. Supervision: P.A.G., S.Y.V., J.F. Visualisation: M.J., M.P. Writing original draft: M.P. Writing– review & editing: M.J., H.S.

**Correct**

Conceptualisation: **N.R.E.S**., H.S. Data curation: M.J., M.P. Formal analysis: M.J., S.H. Investigation: **N.R.E.S**., H.S., M.K.J. Methodology: **N.R.E.S**., H.S. Supervision: P.A.G., S.Y.V., J.F. Visualization: M.J., M.P. Writing original draft: M.P. Writing– review & editing: M.J., H.S.

The original article has been updated.

The authors would like to apologise for any inconvenience caused.

